# Pharmacokinetics and Efficacy of the Spleen Tyrosine Kinase Inhibitor R406 after Ocular Delivery for Retinoblastoma

**DOI:** 10.1007/s11095-014-1399-y

**Published:** 2014-06-07

**Authors:** Eleanor M. Pritchard, Elizabeth Stewart, Fangyi Zhu, Cori Bradley, Lyra Griffiths, Lei Yang, Praveen Kumar Suryadevara, Jiakun Zhang, Burgess B. Freeman, R. Kiplin Guy, Michael A. Dyer

**Affiliations:** 1 Department of Chemical Biology and Therapeutics, St. Jude Children’s Research Hospital, Memphis, Tennessee USA; 2 Department of Developmental Neurobiology, St. Jude Children’s Research Hospital, Memphis, Tennessee USA; 3 Department of Preclinical Pharmacokinetics, St. Jude Children’s Research Hospital, Memphis, Tennessee USA; 4 Department of Ophthalmology, University of Tennessee Health Sciences Center, Memphis, Tennessee USA; 5 Howard Hughes Medical Institute, Chevy Chase, Maryland USA; 6 Department of Developmental Neurobiology, St Jude Children’s Research Hospital, 262 Danny Thomas Place, Memphis, Tennessee 38105 USA

**Keywords:** Ocular drug delivery, R406, Retinoblastoma, Spleen tyrosine kinase

## Abstract

**Purpose:**

Retinoblastoma is a childhood cancer of the retina. Clinical trials have shown that local delivery of broad spectrum chemotherapeutic agents is efficacious. Recent studies characterizing the genomic and epigenomic landscape of retinoblastoma identified spleen tyrosine kinase (SYK) as a promising candidate for targeted therapy. The purpose of this study was to conduct preclinical testing of the SYK antagonist R406 to evaluate it as a candidate for retinoblastoma treatment.

**Methods:**

The efficacy of the SYK antagonist R406 delivered locally in a human orthotopic xenograft mouse model of retinoblastoma was tested. Intraocular exposure of R406 was determined for various routes and formulations.

**Results:**

There was no evidence of efficacy for subconjunctival. R406. Maximal vitreal concentration was 10-fold lower than the minimal concentration required to kill retinoblastoma cells *in vitro*. Dosage of R406 subconjunctivally from emulsion or suspension formulations, direct intravitreal injection of the soluble prodrug of R406 (R788), and repeated topical administration of R406 all increased vitreal exposure, but failed to reach the exposure required for retinoblastoma cell death in culture.

**Conclusion:**

Taken together, these data suggest that R406 is not a viable clinical candidate for the treatment of retinoblastoma. This study highlights the importance of pharmacokinetic testing of molecular targeted retinoblastoma therapeutics.

**Electronic supplementary material:**

The online version of this article (doi:10.1007/s11095-014-1399-y) contains supplementary material, which is available to authorized users.

## Introduction

Retinoblastoma is the most common primary intraocular malignancy of childhood and the third most common pediatric cancer in infants. Each year, approximately 300 cases of retinoblastoma are diagnosed in the United States and 5,000 cases are diagnosed worldwide (1). While mortality is low with aggressive multimodal therapy, partial or full loss of vision occurs in approximately 50% of patients with advanced bilateral retinoblastoma (2). In addition, there are significant late effects of therapy including facial malformations and increased incidence of secondary malignancies (3–5). Locally delivered targeted therapies may maintain high cure rates while improving ocular salvage and vision preservation and reducing late effects of therapy.

Recently, characterization of the genetic and epigenetic landscapes of retinoblastoma revealed increases in expression of the proto-oncogene spleen tyrosine kinase (SYK) (6). Though not expressed in normal human retinas, SYK was found to be upregulated in 100% (82/82) of the retinoblastoma samples and SYK was shown to be required for retinoblastoma cell survival (6). The preclinical candidate SYK inhibitor BAY-61-3606 was efficacious in blocking proliferation of human orthotopic retinoblastoma xenografts *in vivo* (6). In this study, we focused on the SYK inhibitor R406. The orally available prodrug of R406, fostamatinib (R788), has advanced into late-phase clinical trials for oral therapy of autoimmune disorders (7, 8). Fostamatinib is a prodrug that is processed to the active form (R406) in the intestine and previous studies have shown that R406 can induce retinoblastoma cell death in culture (6).

We evaluated *in vivo* efficacy of R406 in an orthotopic xenograft mouse model of retinoblastoma using the approach that was previously successful for nutlin-3a and BAY-61-3606 (6, 11): 1) developing a solution formulation suitable for local delivery using FDA-approved ophthalmic additives; 2) then performing preclinical efficacy and pharmacokinetics studies of subconjunctival R406 in combination with systemic topotecan (R406^oc^/TPT^sys^), following a clinically relevant dose and schedule. We found no evidence of efficacy for R406 delivered using this route and formulation, so we performed *in vitro* assays to estimate the minimal exposure required for caspase-induced retinoblastoma cell death and *in vivo* pharmacokinetics studies to determine the intraocular exposure following a subconjunctival dose of R406 in solution. Comparing *in vitro* pharmacodynamic response with the *in vivo* pharmacokinetic profile, we showed that vitreal exposure of R406 following subconjunctival delivery of R788 was approximately 100-fold lower than the minimal exposure required to kill retinoblastoma cells in culture ( > 1 μM for at least 12 h).

Next, we performed PK studies of alternate formulations of R406 to determine if intraocular exposure following subconjunctival dosing could be increased. Formulation of R406 in higher concentration emulsions or suspensions increased vitreal exposure compared with solution formulation but still did not achieve target exposure, so we expanded our investigation to include alternative routes and prodrugs.

Intravitreal injections of aqueous soluble drugs have been used for retinoblastoma treatment. To determine if R788 (the more water soluble prodrug form of R406) was a candidate for local delivery we examined conversion of R788 to R406 in extracted murine vitreous. After demonstrating that R788 is converted to R406 by phosphatases in the vitreous, we performed pharmacokinetic studies of locally delivered R788. Intravitreal and subconjunctival delivery of R788 failed to achieve target doses of R406. Topical delivery of a lipophilic R406 palmitate salt in eye drops achieved the highest intraocular levels of R406 (~1-2 μM), which were sustainable *via* repeated topical dosing, but exposure was still below our designated therapeutic target.

We examined all feasible delivery routes and formulations for the SYK inhibitor R406 and found that none achieved an intraocular exposure needed to induce retinoblastoma cell death. These data combined with the lack of evidence of efficacy in a preclinical mouse model suggest that R406 is not a viable clinical candidate for retinoblastoma. These data on R788/R406 highlight the importance of careful drug formulation and route selection along with comprehensive pharmacokinetics in preclinical models before moving new therapies into clinical trials.

## Materials and Methods

### Chemicals and Materials

The internal standard OSI-906 ( > 99% purity, batch lot S109103) was purchased from Selleck Chemicals (Houston, TX). R406 phenylsulfonate salt and R788 were purchased from Selleck Chemicals (Houston, TX, USA). LC-MS Chromasolv grade acetonitrile (ACN) was purchased from Fisher Scientific (Loughborough, UK). LC-MS Chromasolv grade formic acid was obtained from Sigma-Aldrich (St. Louis, MO). Milli-Q water as an ultrapure laboratory grade water was used in aqueous mobile phase. Blank murine plasma was obtained from Hilltop Lab Animals, Inc. (Scottdale, PA, USA). Blank murine vitreous was harvested from a mixed population of mice and stored at −80°C until use. All other reagents were of analytical grade or higher. Preparation of R406 free base and palmitate salt are described in the Supplementary Information. For chemical structure of R406 free base, R406 phenylsulfonate salt, R406 palmitate salt and R788, see Fig. S1.

### Formulations

For oral delivery of R788, the target dose of 25 mg/kg R788 was administered by oral gavage as a 4 mM solution in 0.01 N citrate buffer, pH 6 (9). For subconjunctival administration, R788 was prepared at a concentration of 4 mM in 2% PEG-800 and filtered through a 0.22 μm filter prior to use (see Table S1). R788 was also administered subconjunctivally as a suspension at a concentration of 25 mM in 10% 2-hydroxylpropyl-β-cyclodextrin (HPβCD, Sigma-Aldrich). For subconjunctival administration, R406 phenylsulfonate salt was administered at a concentration of 800–900 μM as a cosolvent solution in a formulation of 5% Cremaphor-eL, 0.5% ethanol, 0.2% Tween 80 and 94.3% PBS, filtered through a 0.22 μm filter prior to use (see Table S2). R406 phenylsulfonate salt was also administered subconjunctivally at a dose of 31.4 mM solution in DMSO. R406 free base was administered subconjunctivally at a concentration of 6.5 mM as a 25% castor oil (Sigma-Aldrich) emulsion. This emulsion was prepared by mixing castor oil: ethanol (1:1) solution of R406 (approx. 35 mM), *in vacuo* evaporation of the ethanol, and centrifugation to remove any excess R406, then emulsifying the R406 oil phase in an aqueous phase comprised of Cremaphor-eL, Tween 80 and PBS (final composition of the emulsion: 25% castor oil, 5% Cremaphor-eL, 0.2% Tween-80, 69.8% PBS) by probe sonication. For intravitreal injection, R788 solution was prepared by filtering a 0.12 mM (120 μM) solution of R788 in saline through a 0.22 μm filter. R788 solution was diluted to a concentration 10-fold lower than the compound’s maximum solubility in saline (approx. 1.2-1.4 mM) before administration to mice to better approximate the maximum concentration achievable in a human intravitreal injection. For topical delivery, 25–30 mM suspensions were prepared in 5% HPβCD. R406 or R788 concentration in all formulations was determined by UPLC prior to study (in cases where formulation was filtered before administration, concentration was determined post-filtering) Table I.

**Table 1:** Formulation, administration route and dosing summary for all *in vivo* pharmacokinetic studies

Compound	Administration Route	Formulation	Dose	Total Dose Per Eye (nmol)
R788 (Fostamatinib disodium)	Oral	Solution in citric acid buffer	25 mg/kg	N/A
	Subconjunctival	Co-solvent Solution: 2% PEG in PBS	10 μL of 4 mM per eye	40
	Subconjunctival	Suspension: 10% cyclodextrin in PBS	10 μL of 25 mM per eye	250
	Intravitreal	Saline	5 μL of 120 μM per eye	0.6
R406 phenyl-sulfonate salt	Subconjunctival	Co-solvent Solution: 5% Cremaphor-eL, 0.5% ethanol, 0.2% Tween 80 in PBS	10 μL of 800–900 μM per eye	8−9
	Subconjunctival	DMSO	10 μL of 31.4 mM per eye	314
R406 free base	Subconjunctival	Emulsion: 25–30% castor oil, 5% Cremaphor-eL, 0.2% Tween-80 in PBS	10 μL of 6.5 mM per eye	65
	Topical	Suspension: 5% HPβCD in PBS	2 μL of 25 mM	50
R406 palmitate salt	Topical	Suspension: 5% HPβCD in PBS	2 μL of 25 mM	50
	Topical	Suspension: 5% HPβCD in PBS	2 μL of 40 mM, administered hourly 1, 2, 3 or 4 times	80, 160, 240 or 320

### Efficacy Testing of R406 in a Human Orthotopic Xenograft Mouse Model of Retinoblastoma

**Fig. 1:**
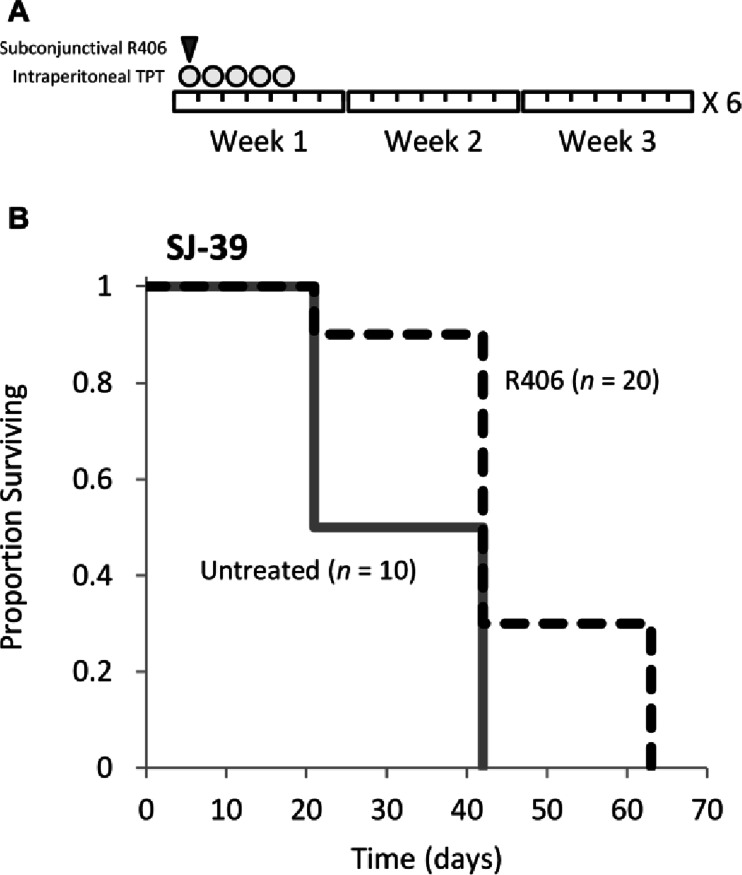
In a human orthoptopic xenograft preclinical mouse model of retinoblastoma subconjunctival R406 does not improve provide efficacy. (**a**) Chemotherapy schedule for 1 course of therapy. Animals were scheduled to complete 6 courses of therapy (18 weeks). (**b**) Kaplan Meier plot of event free survival (EFS) of orthoptopic xenograft mice over time (event defined here as elevated intraocular pressure (IOP > 20) or eye rupture). One group received subconjunctival R406 in a solution formulation (10 μL per eye of approx. 800–900 μM R406 phenylsulfonate salt per dose) in combination with systemic topotecan (TPT) (0.7 mg/kg per dose, i.p.) (*n* = 20) and another group was untreated (*n* = 10). No animals in either group completed the entire 6 courses of treatment; all animals were taken off study by 63 day.

Preclinical testing was carried out in human orthotopic xenograft mice using the methods previously described (10, 11). SJ-39 retinoblastoma cells were injected into the vitreous of 30 immunocompromised mice (J:NU, The Jackson Laboratory (Bar Harbor, ME, USA)): 10 were untreated and 20 received subconjunctival injections of R406 in solution formulation (R406^oc^) and systemic topotecan (TPT^syst^) combination therapy administered over a 5-day course every third week as follows: 10 μL R406 per eye at a concentration of 0.8 - 0.9 mmol (8–9 nanomoles/eye) on day 1, and TPT^syst^ (0.7 mg/kg per dose, i.p.) on days 1 to 5 (see Fig. 1a). Mice were scheduled to receive six courses of therapy (18 weeks total). Before each course of therapy, mice were examined and intraocular pressure (IOP) was read as previously described (10, 11). If intraocular pressure was elevated above normal (IOP > 20) or eye rupture was observed, mice were taken off study and time to event was recorded. At the completion of the study event-free survival (EFS) was analyzed for the R406^oc^/TPT^syst^ treated and untreated groups.

### Bioconversion of R788 to R406 in Mouse Vitreous

Conversion of R788 to R406 was examined in freshly harvested murine vitreous, 2 U/mL bovine alkaline phosphatase in PBS (positive control), or plain PBS (no enzyme, negative control) at 37°C using a modified version of previously described phosphate prodrug conversion protocols (9, 12). Alkaline phosphatase (ALP) from bovine intestinal mucosa was obtained from Invitrogen and was reconstituted and diluted according to the manufacturer’s instructions. Vitreous was harvested from a mixed population of mice, pooled, and kept on ice for 1 - 2 h. The incubation media were pre-warmed to 37°C before the reaction was initiated by addition of 1 mM of R788 in saline (6 μL of R788 in saline into 234 μL of incubation media in a 1.5 mL Eppendorf tube). R788 was diluted into the incubation media at a 1:40 ratio (volume:volume) to mimic the dilution encountered during intravitreal injection in humans (typically 100 μL injection volume into 4–5 mL of human vitreous volume (13, 14). Initial estimated concentration of R788 was 25 μM. At the desired time points (0, 5, 10, 20, 30, 45, 60, and 120 min) an aliquot of 25 μl was collected from each incubation vial and transferred to a 1.5 mL Eppendorf tube, containing 25 μl of acetonitrile to terminate the reaction. 3 samples were tested per time point. Samples were stored at −80°C until quantification.

### Animals

All procedures were approved by the St. Jude Institutional Animal Care and Use Committee and conducted in accordance with the National Institutes of Health guidelines for the care and use of laboratory animals (15). The animal facility is accredited by the American Association for Accreditation of Laboratory Animal Care. For oral gavage and subconjunctival studies, adult female C57BL/6 and B6D2F1/J mice were purchased from The Jackson Laboratory (Bar Harbor, ME, USA). Heterozygous female nudes approximately 11 weeks in age were used for intravitreal injection studies. For topical studies, adult female C57BL/6 mice were purchased from Charles River (Wilmington, MA, USA). Mice were housed in a temperature-controlled room on a normal 12-h light/dark cycle, with free access to water and standard laboratory food.

### PK Study Drug Administration and Sample Collection

For the systemic delivery study R788 was administered as a single bolus dose (25 mg/kg, 10 mL/kg volume) by oral gavage. For the subconjunctival administration study R788 or R406 (10 µL of formulation) was injected into the subconjunctival space of an anesthetized mouse using a Hamilton microliter syringe (Hamilton company, Reno NV). For the intravitreal administration study, R788 (5 μL of 0.15 mM in saline) was administered to each eye *via* direct injection. To perform the intravitreal injection, mice were anesthetized, a small incision between the sclera and the cornea was created with a 13G needle, and the R788 was injected into the vitreous using a Hamilton microliter syringe. For the topical administration study, R406 (2 μL of 25–30 mM suspension) was applied as a drop to the surface of each eye of an anesthetized mouse. All routes, doses and formulations tested are summarized in Table I.

At serial time points (0.5, 1, 2, 4, and 8 h post-injection, *n* = 3 for each time point), blood was collected by terminal cardiac puncture after isoflurane anesthesia. Whole-blood samples were centrifuged immediately at 10,000×*g* for 5 min at 4°C to separate plasma. Once the cardiac puncture and blood collection was completed, the animal was perfused with 10 mL of saline and euthanized by cervical dislocation. Eyes were removed, rinsed in PBS, gently patted dry, then rinsed and dried again. Eyes were dissected and vitreous was collected. Plasma and vitreous samples were put on dry ice immediately after collection and stored at −80°C until analysis. For quantitative analysis methods and assay validation methods and results, see Supplementary Information.

### PK Data Analysis

Plasma and vitreous concentration time data were analyzed using non-compartmental analysis in WinNonlin 6.2 (Pharsight, Mountain View, CA), providing PK parameters including C_max_ (maximum observed concentration), T_max_ (time to reach maximum observed concentration), AUC (area under the concentration time curve) and MRT (mean residence time). AUC and AUMC (area under the concentration time first moment curve) values were estimated with the linear trapezoidal method, with parameters calculated using mean concentration values at each time point. To obtain the mean concentrations at time points with data below the lower limit of quantitation (BLOQ), BLOQ observations were replaced with a value of ½ the lower limit of quantitation (LLOQ) if ≥ 2/3rds of the observed concentrations were above the LLOQ; otherwise, the mean concentration was treated as missing.

## Results

### Preclinical Testing of R406 in Mouse Models of Retinoblastoma

Previous studies have shown that R406 and BAY61-3606 can induce caspase-mediated cell death of retinoblastoma cells *in vitro* (6). *In vivo* administration of BAY61-3606 showed efficacy in human orthotopic xenografts models (6) but R406 was not tested *in vivo* in that previous study. Since R406 is the SYK inhibitor farthest along in clinical development (Phase 3), and therefore the most likely compound to be tested clinically against retinoblastoma, we decided to focus upon its evaluation. In order to test the efficacy of R406 *in vivo*, we developed a solution formulation using FDA approved adjuvants for ophthalmic applications (11). The maximum concentration of R406 in solution was approximately 900 μM and R406 was chemically stable in formulation for 48 h at room temperature (Table S3 and Fig. S2). Next, we performed *in vivo* efficacy studies using the solution formulation of R406 in combination with systemic topotecan (standard for our preclinical evaluation of new retinoblastoma agents (11) following a clinically relevant dose and schedule as was previously done for nutlin-3a and BAY-61-3606 (6, 11). SJ-39 retinoblastoma cells were injected into the vitreous of 30 immunocompromised mice and animals were randomized into two treatment groups: 20 received subconjunctival injections of R406 in solution formulation (R406^oc^) and systemic topotecan (TPT^syst^) combination therapy administered over a 5-day course every third week (see Fig. 1a) and 10 were untreated. Animals were scheduled to receive 6 courses of treatment (18 weeks total), but no animal completed the entire 6 courses. All animals were taken off study by 63 days due to elevated intraocular pressure (IOP > 20) or eye rupture. Event free survival (EFS) was analyzed for the two groups (Fig. 1b). Log-rank test showed that there was a statistically significant difference in the distribution of time to event between the two groups (*p* = 0.005), and the treated group had a longer EFS than the untreated group. The proportion of event free survivals at 21 days were 50% and 90% for the untreated and treated group, respectively; at 42 days the EFS proportions were 0% and 30% for the untreated and treated group, respectively. Median event free survivals were 32.5 days and 42 days for untreated and treated group, respectively. This is very similar to our historical studies with TPT^syst^ alone. Though there is a slight improvement in outcome for the R406^oc^/TPT^syst^ treated group, no animals survived beyond 63 day, suggesting R406 delivered with this route and formulation does not substantially delay disease progression.

#### Pharmacokinetics of Subconjunctival R406 Ocular Solution in Mice

**Fig. 2:**
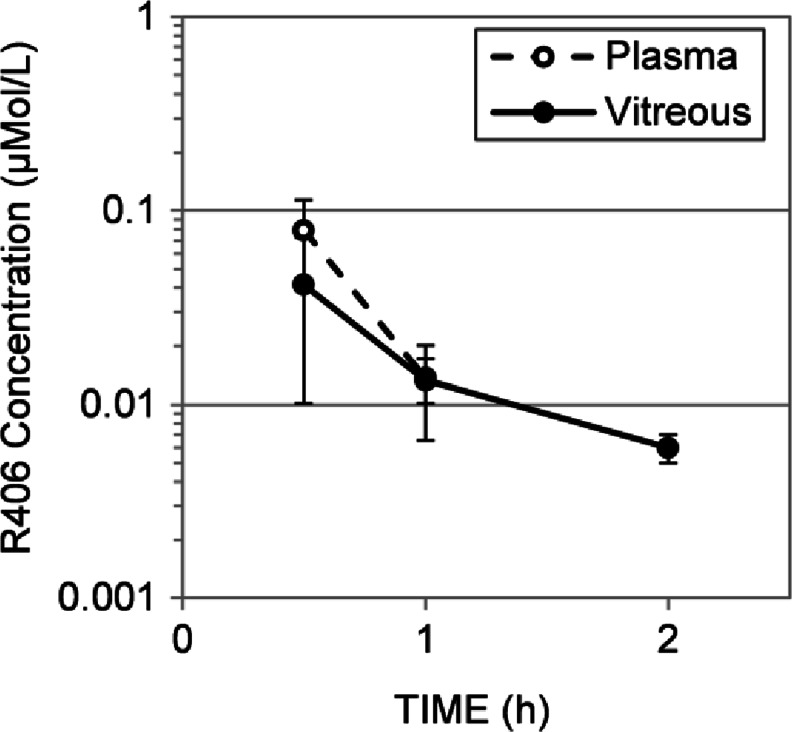
Pharmacokinetic behavior of R406 administered as in the efficacy study. Concentration of R406 measured at 0.5, 1.0, 2.0, and 4.0 h in the plasma (dashed line, empty circles) and vitreous (solid line, filled circles) following subconjunctival delivery of R406 salt in cosolvent solution (10 μL per eye of 880 μM R406 phenylsulfonate salt).

**Fig. 3:**
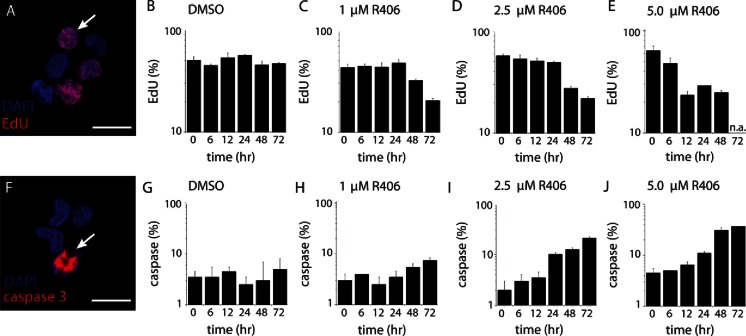
Cellular pharmacodynamic study defining target time concentration curve RB355 cells in culture are exposed to DMSO, 1, 2.5 and 5 μM R406 for 0, 6, 12, 24, 48, or 72 h. (a to e) EdU staining of RB355 cells treated with 0 (DMSO), 1, 2.5, or 5 μM R406 for 0, 6, 12, 24, 48, or 72 h. (f to j) Caspase staining of RB355 cells treated with 0 (DMSO), 1, 2.5 or 5 μM R406 for 0, 6, 12, 24, 48, or 72 h.

To determine if the lack of evidence of efficacy of subconjunctival R406 in a solution formulation was due to suboptimal ocular exposure, we performed a pharmacokinetic experiment. Briefly, 10 μl of R406 solution formulation was delivered to each eye of 15 mice and the plasma and vitreous were harvested at different time points (0.5, 1, 2, 4, and 8 h). The concentration of R406 was measured at each time point in each tissue and the concentration of drug was plotted as a function of time (Fig. 2). The 4 h and 8 h data are not shown, as the majority of R406 concentrations in these samples were BLOQ. These data show that systemic exposure (plasma) was low following subconjunctival delivery of R406 as expected (Fig. 2). However, exposure of R406 in the vitreous failed to reach pharmacologically significant thresholds (Fig. 2). Importantly, the maximal concentration achieved (0.05 μM) was well below the concentration required to kill the most sensitive retinoblastoma cell line (RB355 in culture, Fig. S3). RB355 cells in culture were treated with DMSO, 1, 2.5, or 5 μM of R406 for varied exposure durations (0, 6, 12, 24, 48 and 72 h). For the cells in the 2.5 and 5 μM treatment groups, the proportion of activated caspase 3+ cells measured by immunostaining increased with increasing exposure duration. Incorporation of 5-ethynyl-2′-deoxyuridine (EdU) was used to detect DNA synthesis. The proportion of cells that incorporated EdU in the 5 μM treatment groups decreased with increasing exposure time (Fig. 3e). In contrast, cells treated with 1 μM R406 did not show any increase in caspase 3+ activation compared to untreated cells or cells treated with DMSO for any exposure time tested up to 48 h (Fig. 3f to j). Viability of RB355 cells in culture is reduced to 50% of the viability of DMSO treated cells when exposed to 2.5 μM R406 for an exposure duration between 24 and 48 h or 5 μM R406 for an exposure duration between 12 and 24 h. There is no reduction in viability compared to DMSO treated controls for RB355 cells in culture treated with 1 μM R406 for up to 24 h exposure, and at longer exposure durations (48 – 72 h), viability only drops by approximately 20% (Fig. S3). Comparing the *in vivo* pharmacokinetics (Fig. 2) and *in vitro* pharmacodynamics data (Fig. 3, Fig. S3) suggests that the lack of evidence of efficacy observed in the preclinical study (Fig. 1b) was due to insufficient intraocular exposure of R406 from the solution formulation.

#### Pharmacokinetics of R406 Emulsion, Suspension, and Drops

**Fig. 4:**
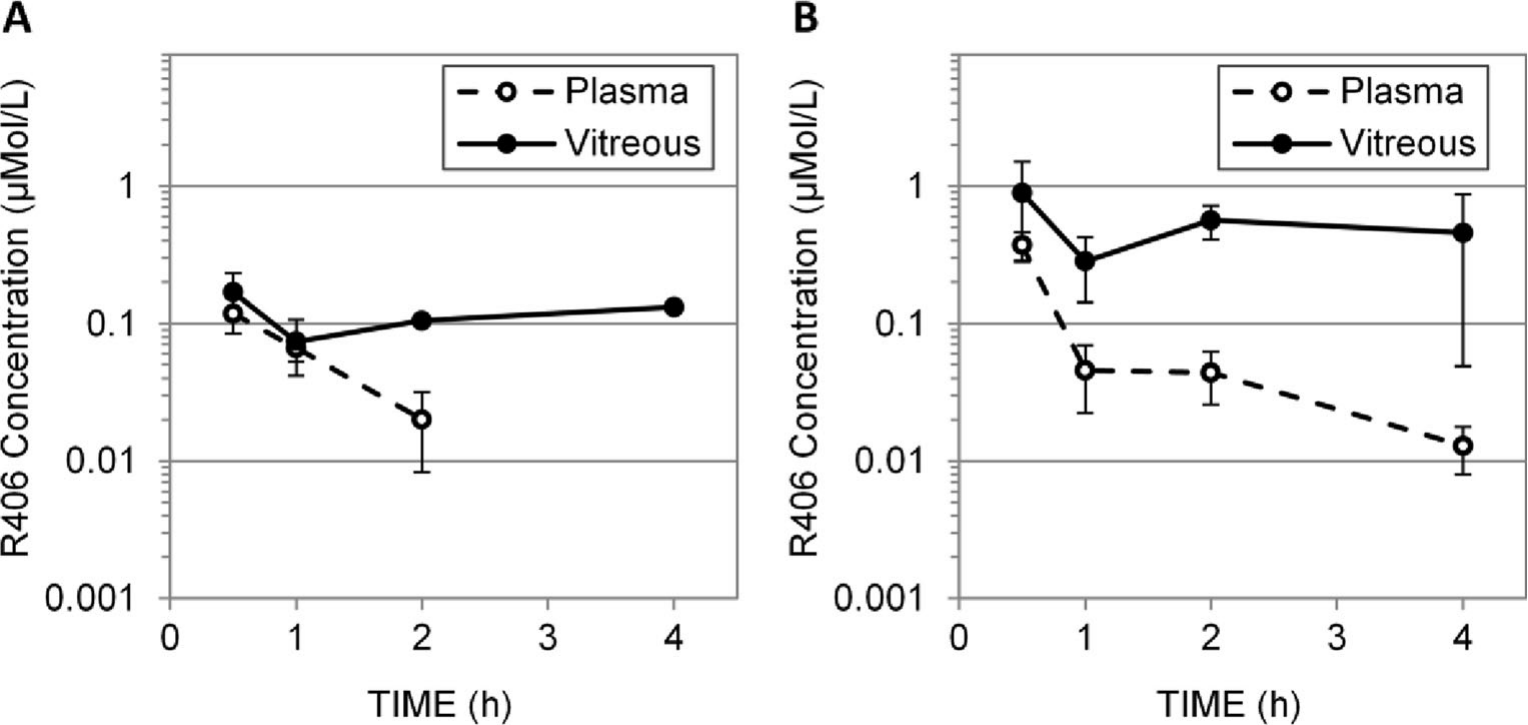
Pharmacokinetics of R406 following subconjunctival administration. Concentration of R406 measured at 0.5, 1.0, 2.0, and 4.0 h in the plasma (dashed line, empty circles) and vitreous (solid line, filled circles) following (**a**) subconjunctival delivery of R406 free base in a 25–30% castor oil emulsion (10 μL per eye of 6.5 mM R406 free base). (**b**) subconjunctival delivery of R406 in DMSO (10 μL per eye of 31.4 mM R406 phenylsulfonate salt). Neither formulation achieves the target drug concentration in the eye.

In order to increase the concentration of R406 delivered to the eye from subconjunctival injection, we developed an emulsion formulation using castor oil and other FDA approved adjuvants for ophthalmic emulsions (11). Using this approach, we achieved a concentration of 6.5 mM of R406 in emulsion and the emulsion was stable for 48 h at room temperature (Fig. S10, Tables S12 and S13). This provided a formulation with 7-fold increased R406 concentration compared to the solution formulation. We performed a pharmacokinetic experiment after delivery of 10 μl of R406 emulsion as described above for the R406 solution (Fig. 4a). As a control, we used a high concentration (31.4 mM) of R406 in DMSO. While it is not clinically feasible to deliver R406 in DMSO and DMSO might disrupt biological membranes causing an overestimate of vitreous exposure, it provides a useful benchmark for peak concentration of R406 achievable from subconjunctival delivery (Fig. 4b). The R406 emulsion and R406 in DMSO both led to a significant increase in maximal vitreal concentration and overall exposure but neither reached the targeted efficacious exposure (Fig. 4a and b, Table II). Subconjunctival R406 suspension was not evaluated, as the DMSO control indicated the maximum intraocular concentration that could be reached for R406 from this route was approximately 1 μM.

**Table II:** Summary of vitreous and plasma pharmacokinetic parameters after a single administration (systemic delivery/oral route or local delivery/subconjunctival injection) of R406 or R788 to mice

	R788 (Fostamatinib Disodium)			R406 Phenylsulfonate Salt		R406 Free Base
Route	Oral	Local	Local	Local	Local	Local
Formulation	Buffered Solution	Co-solvent Solution	Suspension	Co-solvent Solution	DMSO	Castor Oil Emulsion
Concentration (mM)	4	4	25	0.88	31.4	6.5
Dose (nmol per eye, except where noted)	25 mg/kg	40	250	8.8	314	65
Plasma PK Parameters:						
C_max_ (nM)	2,009.7	574.2	2,906.6	79	370.2	118.6
T_max_ (h)	0.5	0.5	0.5	0.5	0.5	0.5
AUC_last_(nM∙ h)	1,500	306	1,800	42.9	298	119
Dose normalized AUC (nM∙h/nmol)	1.9	3.8	3.6	2.4	0.5	0.9
T_last_(h)	4	1	4	1	4	2
Vitreous PK Parameters:						
C_max_ (nM)	164 ± 68	969 ± 39.8	267 ± 76.4	41.3 ± 18	892 ± 350	169 ± 35.9
T_max_ (h)	0.5	0.5	0.5	0.5	0.5	0.5
AUC_last_(nM∙h)	125	724	456	33.7	1,960	430
Dose normalized AUC (nM∙h/nmol)	0.2	9.1	0.9	1.9	3.1	3.3
T_last_(h)	2	4	4	2	4	4
MRT_0-4h_ (h)	0.772	0.861	1.69	0.782	2.02	2.19
Ratio Vitreous AUC_last_/Plasma AUC_last_	0.08	NA	0.3	NA	6.6	NA

**Fig. 5:**
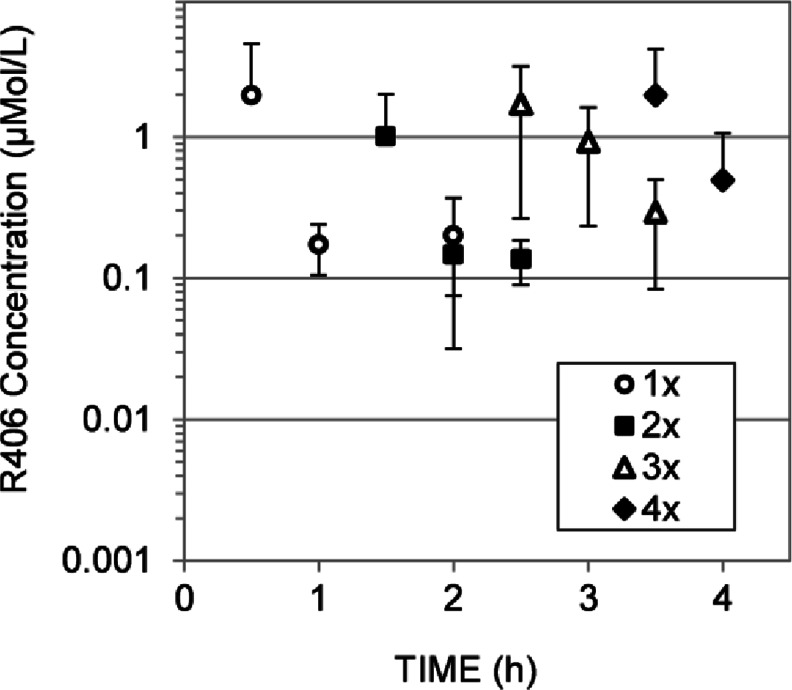
Pharmacokinetics of R406 following topical administration. Intraocular R406 concentration measured at 0.5, 1, and 2 h post dosing with one, two, three, or four topical administrations of R406 palmitate salt in suspension (2 μL per eye of 40 mM R406 palmitate salt in 5% HPβCD per dose). Vitreous collected 0.5, 1, and 2 h after the latest dose. *N* = 3-5. Error bars represent standard deviations (where error bars are not shown they fall into background).

Previous work has shown that ocular absorption of a drug *via* the topical route (drops applied to the cornea) can be enhanced by increasing its lipophilicity (16). To evaluate the topical route for R406 delivery, we tested the intraocular concentration after a single topical dose of R406 free base or an R406 palmitate salt. R406 levels in the eye were 0.1 ± 0.06 μM at 0.5 h and 0.1 ± 0.1 μM at 1 h post dosing with R406 free base drops, and 0.3 ± 0.3 μM at 0.5 h and 0.3 ± 0.2 μM 1 h post dosing with R406 palmitate salt drops. The R406 palmitate salt achieved higher intraocular concentrations than the R406 free base, but the difference between average intraocular concentration at both time points was not statistically significant (*t*-test, p-value for 0.5 h = 0.3, *p*-value for 1 h = 0.2). No detectable drug was present in the plasma samples (data not shown). The advantage of ocular drops is that repeat daily dosing can be readily achieved. To determine if repeated dosing of the R406 palmitate salt drops could increase intraocular exposure, we dosed mice up to four times with a 40 mM R406 palmitate 5% cyclodextrin suspension and measured the vitreal concentration of R406 at 0.5, 1, and 2 h after the last of 1, 2, 3, or 4 hourly doses (Fig. 5). While exhibiting high variability (coefficient of variation (CV) > 40% at nearly all observations), mean intraocular concentration-time profiles during the period of time 0.5 to 2 h post-dosing were similar for repeated doses, with some increase in the mean value at the 1 h post dosing time point for 3 or 4 doses compared with 1 or 2 doses. A vitreous concentration of ~1-2 μM is sustained for 1.5 h after each application of eye drops, demonstrating that R406 concentrations above 1 μM could be sustained using the topical route. However the maximal concentration achieved is still below the targeted concentration.

### Intravitreal Injection of R788

**Fig. 6:**
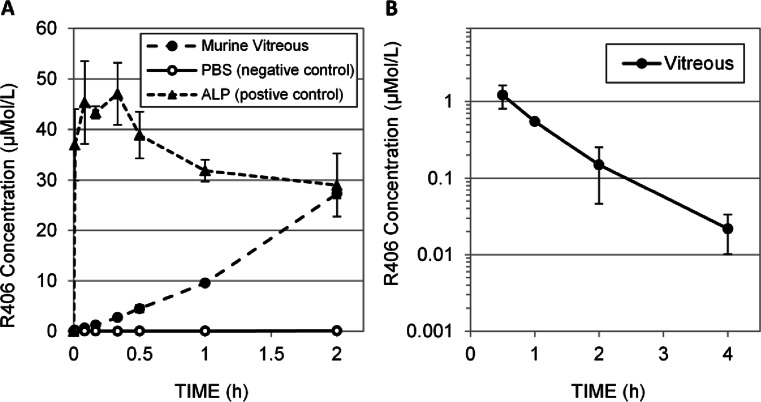
Pharmacokinetics of R406 following intravitreal administration of R788. (**a**) Bioconversion of R788 to R406 in freshly harvested, pooled murine vitreous (filled circles), PBS alone (empty circles), and PBS with 2U/mL alkaline phosphate enzyme (ALP) (triangles). *N* = 3 samples per incubation media. Error bars represent standard deviations (where error bars are not shown they fall into background). (**b**) Concentration of R406 measured at 0.5, 1.0, 2.0, and 4.0 h in the vitreous (blue, squares) following intravitreal injection of R788 in saline (5 μL per eye of 120 μM R788). R406 levels BLOQ in all plasma samples. Administration of maximal dose of R788 fails to provide target concentrations of R406 for sufficient duration.

Previously, dexamethasone phosphate prodrugs have been successfully administered as subconjunctival injections, resulting in high concentrations of dexamethasone in the vitreous (17, 18). This suggests phosphatases are present in ocular tissues, but phosphatase activity in extracted vitreous has not been characterized. To determine if R788 can be converted to R406 in the mouse eye, we incubated fresh murine vitreous with R788 and measured the accumulation of R406 over time (Fig. 6a). As a negative control, we used PBS; as a positive control, we used PBS with alkaline phosphatase (ALP) (2 U/mL concentration). R788 was converted to R406 (100% conversion within 2 h) in extracted mouse vitreous suggesting that the prodrug can be effectively delivered directly to the eye. High variability in concentration and apparent concentration decrease at later time points in the ALP positive control samples (Fig. 6a) may be related to R406’s solubility limitations.

**Fig. 7:**
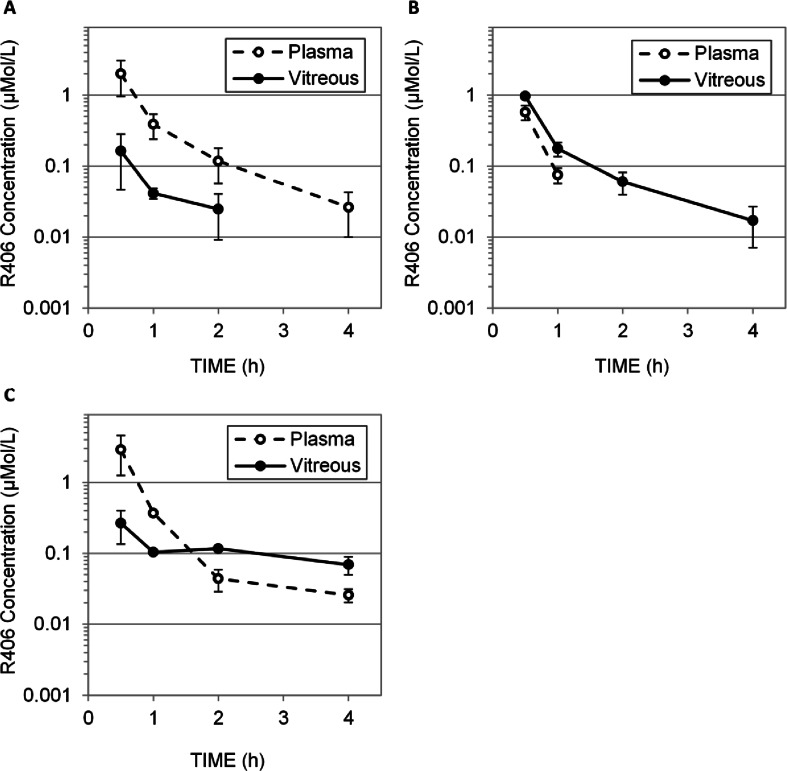
Pharmacokinetics of R406 following administration of R788 by several routes. Concentration of R406 measured at 0.5, 1.0, 2.0, and 4.0 h in plasma (dashed line, empty circles) and vitreous (solid line, filled circles) following (**a**) oral delivery of R788 (25 mg/kg R788 in citric acid buffer) (**b**) subconjunctival delivery of R788 cosolvent solution (10 μL per eye of 4 mM R788). (**c**) subconjunctival delivery of R788 suspension (10 μL per eye of 25 mM R788). No route achieves the targeted drug exposure.

There are now several examples of direct injection of chemotherapy or biological agents into the vitreous for the treatment of retinoblastoma in patients (19–21). Direct intraocular administration of R788 provides maximal R406 concentration of 1 μM at 0.5 h post dosing (Fig. 6b, the earliest time point measured). Systemic exposure is lower following intravitreal injection, but the vitreous concentration-time curve is similar to that following subconjunctival injection of R788 in solution formulation (Fig. 7b). Oral delivery of R788 and subconjunctival injection of R788 in suspension show no improvement in overall exposure compared to intravitreal injection of R788 (Fig. 7a and c).

## Discussion

### Local Delivery of Molecular Targeted Therapy for Retinoblastoma

Due to the unique physiology of the eye, numerous routes of delivery are available. Systemic routes can be used for ocular therapeutics, but are typically limited by the Blood Retinal Barrier (BRB), which drives up doses needed to reach efficacious concentrations, thus causing increased systemic toxicity (22–24). Therefore local delivery, which can drastically reduce systemic exposure while simultaneously increasing ocular exposure, is an attractive option.

Local ocular delivery routes generally fall into four categories: topical (transcorneal), periocular (transcleral), intravitreal (direct injection), and intra-arterial infusion (21, 25, 26). Though multiple delivery routes are available for retinoblastoma therapeutics, all routes are not amenable to all compounds. Some chemotherapy drugs like topotecan effectively cross the BRB, with equivalent intraocular PK profiles from either systemic or local topotecan delivery (10, 27). In contrast, carboplatin (28–30) and nutlin-3a (11, 31) cannot be administered systemically due to insufficient penetration across the BRB and in these cases subconjunctival injection has been significantly more effective. This contrast highlights the importance of utilizing PK to direct delivery route selection for ocular therapeutics.

We examined all feasible routes of delivery to the eye including oral administration of R788 (the conventional route of administration in clinical RA trials), subconjunctival delivery of R406, and topical delivery of R406 and a more lipophilic R406-palmitate salt. Bioconversion of R788 to R406 in pooled extracted murine vitreous supported testing local administration of the phosphate prodrug, so subconjunctival administration of R788 and intravitreal injection of R788 was also evaluated.

To achieve efficacy as a retinoblastoma therapeutic, R406 must reach therapeutically effective intraocular exposure. In preclinical mouse models of retinoblastoma, subconjunctival R406 did not result in evidence of efficacy, and our studies suggest this can be attributed to insufficient intraocular exposure. Though local administration routes improved intraocular exposure relative to systemic delivery (Fig. 6), none of the subconjunctival or intravitreal formulations tested provided a vitreous C_max_ above 1 μM, which our *in vitro* studies suggest would be required to drive efficacy even at prolonged exposure times (*i.e*., up to 72 h) (Fig. 3). Only topical delivery of the R406 palmitate salt in cyclodextrin drops provided a vitreous C_max_ above 1 μM, which can be sustained *via* repeated dosing. The topical route achieved higher intraocular concentrations of R406 than any other route tested and has the advantages of non-invasiveness and ease of administration of repeated doses. No chemotherapy drugs are currently administered topically, but these results highlight the need for further exploration of this route for retinoblastoma therapeutics.

### R406 Ocular Pharmacokinetics

Our results suggest that the primary drivers of ocular PK for R406 are (1) aqueous solubility (2) lipophilicity and (3) dissolution behavior. In PBS, the maximum solubility of R788 is 1.4 mM, the maximum solubility of the R406 phenylsulfonate salt is 0.4 μM and R406 free base is insoluble (below the limit of quantitation). Lipophilicity of the compounds tested follows the order (least lipophilic to most): R788 < R406 phenylsulfonate salt < R406 free base, while aqueous solubility follows the order (lowest solubility to highest): R406 free base < R406 phenylsulfonate salt < R788. At similar total doses, the more water soluble prodrug R788 in cosolvent solution (40 nmol total dose per eye) achieves a maximum vitreous concentration 6-fold higher than the water insoluble R406 free base in emulsion (65 nmol total dose per eye) (Figs. 6b and 3a, Table II), which suggests aqueous solubility may control maximal equilibrium ocular drug concentrations. R406 administered at a high dose in DMSO achieves a comparable vitreous C_max_ to R788 cosolvent formulation (Figs. 3b and 6b, Table II), suggesting that the upper limit of compound solubility in vitreous is driven by solubility of the converted R406. A 6.25-fold increase in dose of locally administered R788 does not increase vitreous C_max_ or vitreous exposure, but dramatically increases systemic exposure (Table II). This suggests rapid clearance occurs once the maximum vitreous concentration is reached, and that increasing the amount of drug administered to the subconjunctival space will not increase vitreous C_max_ beyond this limit. When comparable total doses of R788 and R406 salt are administered locally (250 and 314 nmol total per eye, respectively), the R406 salt increases vitreous exposure and decreases plasma exposure, achieving a ratio of vitreous AUC to plasma AUC 22-fold higher than the more water-soluble R788 in suspension (Table II). This suggests that when local dose is sufficient, R406 phenylsulfonate salt exhibits greater retention at the injection site due to its limited water solubility, while the more water soluble prodrug diffuses more readily from the injection site into systemic circulation. R406 phenylsulfonate salt and R788 have very similar molecular weights (628.6 and 624.4, respectively), suggesting that their differences in lipophilicity, solubility and dissolution are driving PK.

Ocular mean residence times (from shortest to longest) were as follows: R788 systemic/oral ≈ R406 salt local solution < R788 local solution (all less than 1 h) < R788 local suspension < R406 salt local DMSO < R406 base local emulsion (Table II). The emulsion and the R406 salt in DMSO exhibit the longest mean residence times (MRT ≥ 2 h). The R406 emulsion may form a drug depot in the subconjunctival space, analogous to the use of intramuscular injections of oil carriers and suspensions in delivery of long acting injectable (LAI) antipsychotics where slow diffusion of the drug ester out of the oil phase and into the blood stream prolongs the maintenance of therapeutic drug concentrations (32). When added to buffer, R406 salt in DMSO precipitates out of solution (see Supplement). The solution of R406 salt in DMSO may therefore be functionally comparable to the long-acting injectable formulation of olanzapine, which is injected into the gluteal muscle as a suspension of micron sized crystals of olanzapine and pamoic acid in aqueous solution, providing sustained release (2–4 weeks) as the salt gradually dissolves and diffuses into systemic circulation (33). If R406 salt precipitates out of DMSO in the subconjunctival space, depot formation will lead to re-dissolution of the R406 precipitate sustaining release into the eye and limiting rapid clearance from the injection site. This interpretation is consistent with the rapid clearance and high systemic exposure observed when a relatively high concentration of R788 in suspension is administered locally.

In the case of subconjunctival delivery of R406, increasing compound lipophilicity improves the ratio of vitreous AUC to plasma AUC, but an increase in drug lipophilicity may not improve ocular PK overall if there is a trade-off in aqueous solubility: the most lipophilic of the compounds tested (R406 free base), reaches relatively low intraocular concentrations compared with other local formulations, while the least lipophilic (R788) has the shortest MRT. Compare these results to the previous success with local delivery of nutlin-3a (AUC ratio of vitreous to plasma for subconjunctival administration = 28.6), which has a water solubility of approximately 40 μM (100-fold higher than R406 phenylsulfonate salt) and a higher logP than any of the compounds tested in this study. Our current understanding of subconjunctival administration suggests that successful delivery depends on optimizing a three-way balance between aqueous solubility, lipophilicity, and dissolution because these properties impact absorption across barriers, tissue partition, maximum concentration achievable in vitreous, retention at the injection site, and ratio of compound diffusing out systemically relative to the amount absorbed into the eye. Because inverse relationships may exist amongst solubility, lipophilicity, and dissolution, it is unlikely that any one of those properties can be optimized individually; improvements in one property will need to be weighed against potential trade-offs in another.

### Targeting SYK/BCL2 in Retinoblastoma

Despite the failing of R406 as a retinoblastoma clinical candidate, SYK remains a promising target as there are many other small molecule SYK inhibitors with diverse physiochemical properties to evaluate as retinoblastoma therapeutics (34–38). Previous studies of SYK inhibition have implicated a number of downstream signaling molecules, including the Bcl-2 family of proteins as mediators of the SYK survival signal (35, 39). This suggests that some of the small molecule Bcl-2 inhibitors currently being developed for other cancer, such as obatoclax and TW-37 (40) may also prove to be effective therapeutic agents in retinoblastoma.

### Conclusion

The SYK inhibitor R406, currently in clinical development for rheumatoid arthritis, can induce caspase-mediated cell death of retinoblastoma cells in culture. Subconjunctival administration of R406 failed to provide any evidence of improvement in tumor response in preclinical models of retinoblastoma. We found that vitreal exposure following subconjunctival delivery of the R406 solution formulation was below the exposure required to induce caspase-mediated cell death of retinoblastoma cells in culture. We developed emulsion and suspension formulations of R406, which increased the vitreal exposure of R406, but still failed to reach the target exposure. The R788 prodrug was efficiently converted to R406 in extracted vitreous and *in vivo*, but following direct intravitreal injection of R788, R406 exposure was still insufficient. Topical delivery of R406-palmitate achieved intraocular concentrations above 1 μM, which was sustainable *via* repeated dosing. Though local delivery *via* the subconjunctival, topical and intravitreal routes improved vitreous exposure compared with oral delivery, vitreous exposure from all routes and formulations tested was not within the range required to kill retinoblastoma cells in culture. Therefore, the preclinical models strongly suggest that R406 is not a viable clinical candidate for retinoblastoma. Future efforts may focus on combining R406 with other agents, testing other SYK inhibitors with different physiochemical properties that may improve intraocular pharmacokinetics, or targeting other proteins in the pathway.

## Electronic Supplementary Materials

Below is the link to the electronic supplementary material.

ESM 1 (DOCX 361 kb).

## Abbreviations

ACN: Acetonitrile
ALP: Alkaline phosphatase
AUC: Area under the curve
BLOQ: Below limit of quantitation
BBB: Blood brain barrier
BRB: Blood retinal barrier
CV: Coefficient of variation
DMSO: Dimethyl sulfoxide
EdU: 5-Ethynyl-2’-deoxyuridine
HPβCD: 2-Hydroxylpropyl-β-cyclodextrin
LLOQ: Lower limit of quantitation
NCA: Non-compartmental analysis
PK: Pharmacokinetics
PBS: Phosphate buffered saline
SYK: Spleen tyrosine kinase
