# Supplementary material for: Pharmacokinetics and Efficacy of the Spleen Tyrosine Kinase Inhibitor R406 after Ocular Delivery for Retinoblastoma
Source: Pharm Res. 2014 Jun 7;31(11):3060–72. doi: 10.1007/s11095-014-1399-y (PMC4213378; doi:10.1007/s11095-014-1399-y)
Supplement: Supplementary file 1 — (DOCX 361 kb). [file 11095_2014_1399_MOESM1_ESM.docx]

**Supplementary Information**

**
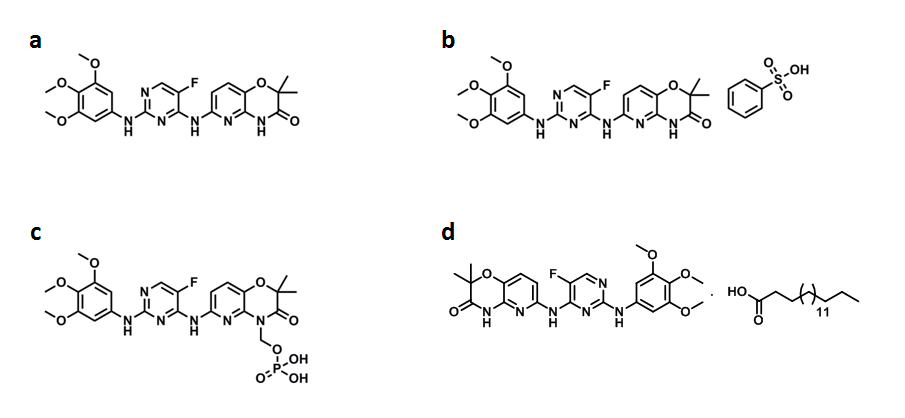
**

**Figure S1.** Chemical structures of (a) R406 free base (b) R406 phenylsulfonate salt (c) R788 (d) R406 palmitate salt

**Preparation of R406 Free Base and R406 Palmitate Salt**

R406 Free Base was prepared as follows: to a solution of R406 phenylsulphonate salt (100 mg) in ice cold water (30 mL) was added 1N NaOH solution, dropwise, until the pH reached 8-9. The resulting mixture was diluted with bicarbonate solution (saturated aq., 20 mL) and extracted with CHCl_3_ (3 x 50 mL). The combined organic layers were dried over anhydrous Mg_2_SO_4_, evaporated under reduced pressure, and purified chromatographically on basic alumina using CHCl_3_/MeOH as eluent to afford R406 free base as a pale white solid with >95% purity by UPLC. R406 palmitate salt was prepared as follows: to a solution of R406 free base (0.045 mg, 0.096 mmol) in ethanol (10 mL) was added a solution of palmitic acid (0.123 g, 0.478 mmol, 5 eq.) in ethanol (3 mL). The resulting mixture was stirred at room temperature for 20 min giving a suspension. This suspension was heated to reflux at which point water (0.8 mL) was added drop wise. After maintaining reflux for 2 h, acetone (0.5 mL) was added to provide a clear solution. Heating was continued for additional 30 min then the solution was cooled to ambient temperature and the solution evaporated *in vacuuo*. The resulting solid was dissolved in diethyl ether (100 mL) and stirred at room temperature for 24 h. After filtration to remove excess palmitic acid the solution was evaporated *in vacuuo* and the resulting residue was dried overnight *in vacuuo* to yield the palmitate salt of R406 as a pale white solid >95% purity

**Table S1.** Matrix to Create Co-solvent Solution Formulation of R788

| Trial Number | % Tyloxapol | %  PEG-8000 | % EtOH | % Glycerin | %  Cremaphor-eL | % PPG | % pPPG | %  Tween-80 | %  PBS | R788 Solubility (mM) |
| --- | --- | --- | --- | --- | --- | --- | --- | --- | --- | --- |
| 1 | 0.1 |  |  |  |  |  |  |  | 99.9 | 1.23 |
| 2 |  | 2 |  |  |  |  |  |  | 98 | **3.56** |
| 3 |  |  | 0.5 |  |  |  |  |  | 99.5 | 1.01 |
| 4 |  |  |  | 3 |  |  |  |  | 97 | 1.13 |
| 5 |  |  |  |  | 5 |  |  |  | 95 | 1.43 |
| 6 |  |  |  |  |  | 10 |  |  | 90 | 1.13 |
| 7 |  |  |  |  |  |  | 15 |  | 85 | 1.53 |
| 8 |  |  |  |  |  |  |  | 4 | 96 | 1.71 |
| 9 |  |  |  |  |  |  |  |  | 100 | 1.24 |
| 10 |  |  |  |  | 5 |  |  | 4 | 91 | 1.51 |
| 11 |  |  |  |  |  |  | 15 | 4 | 81 | 1.84 |
| 12 |  |  |  |  | 5 |  | 15 |  | 80 | 2.15 |
| 13 |  |  |  |  | 5 |  | 15 | 4 | 76 | 2.64 |
| 14 |  | 2 |  |  | 5 |  | 15 | 4 | 74 | 3.47 |

%: percent; PEG-8000: polyethylene glycol 8000; EtOH: ethanol; Cremaphor-EL: polyoxyl 35 castor oil; PPG: propylene glycol; pPPG: polypropylene glycol; PBS: Phosphate Buffered Saline; mM: millimolar

**Table S2.** Matrix to Create Co-solvent Solution Formulation of R406 Phenylsulfonate Salt

| Trial Number | % Tyloxapol | %  PEG-8000 | % EtOH | % Glycerin | %  Cremaphor-eL | % pG | % pPG | %  Tween-80 | %  PBS | R406 Solubility (mM) |
| --- | --- | --- | --- | --- | --- | --- | --- | --- | --- | --- |
| 1 | 0.1 |  |  |  |  |  |  |  | 99.9 | 11.7 |
| 2 |  | 2 |  |  |  |  |  |  | 98 | 0.0 |
| 3 |  |  | 0.5 |  |  |  |  |  | 99.5 | 0.0 |
| 4 |  |  |  | 3 |  |  |  |  | 97 | 0.4 |
| 5 |  |  |  |  | 5 |  |  |  | 95 | 170.1 |
| 6 |  |  |  |  |  | 10 |  |  | 90 | 0.4 |
| 7 |  |  |  |  |  |  | 15 |  | 85 | 15 |
| 8 |  |  |  |  |  |  |  | 0.2 | 99.8 | 399.2 |
| 9 |  |  |  |  |  |  |  |  | 100 | 0.4 |
| 10 |  | 2 |  |  |  |  |  | 0.2 | 97.8 | 0.4 |
| 11 |  |  | 0.5 |  |  |  |  | 0.2 | 85.8 | 0.6 |
| 12 |  |  |  | 3 |  |  |  | 0.2 | 96.8 | 1.0 |
| 13 |  |  |  |  | 5 |  |  | 0.2 | 94.8 | 178.0 |
| 14 |  |  |  |  |  | 10 |  | 0.2 | 99.8 | 0.9 |
| 15 |  |  |  |  |  |  | 15 | 0.2 | 85.8 | 31.4 |
| 16 |  | 2 | 0.5 |  |  |  |  |  | 97.5 | 1.5 |
| 17 |  | 2 |  | 3 |  |  |  |  | 95 | 0.4 |
| 18 |  | 2 |  |  | 5 |  |  |  | 93 | 144.0 |
| 19 |  | 2 |  |  |  | 10 |  |  | 88 | 1.4 |
| 20 |  | 2 |  |  |  |  | 15 |  | 83 | 22.9 |
| 21 |  |  | 0.5 | 3 |  |  |  |  | 96.5 | 0.4 |
| 22 |  |  | 0.5 |  | 5 |  |  |  | 94.5 | 183.9 |
| 23 |  |  | 0.5 |  |  | 10 |  |  | 89.5 | 0.0 |
| 24 |  |  | 0.5 |  |  |  | 15 |  | 84.5 | 18.4 |
| 25 |  |  |  | 3 | 5 |  |  |  | 92 | 183.9 |
| 26 |  |  |  | 3 |  | 10 |  |  | 87 | 0.4 |
| 27 |  |  |  | 3 |  |  | 15 |  | 82 | 18.2 |
| 28 |  |  |  |  | 5 | 10 |  |  | 85 | 161.5 |
| 29 |  |  |  |  | 5 |  | 15 |  | 80 | 138.0 |
| 30 |  |  |  |  |  | 10 | 15 |  | 75 | 23.3 |
| 31 |  |  |  |  | 5 |  |  | 0.2 | 94.8 | 689.9 |
| 32 |  |  | 0.5 |  | 5 |  |  | 0.2 | 94.3 | **830.9** |
| 33 |  |  | 0.5 | 3 | 5 |  |  | 0.2 | 91.3 | 773.9 |

%: percent; PEG-8000: polyethylene glycol 8000; EtOH: ethanol; Cremaphor-EL: polyoxyl 35 castor oil; PPG: propylene glycol; pPPG: polypropylene glycol; PBS: Phosphate Buffered Saline; µM: micromolar

**R406 stability in cosolvent solution**: Precipitation of R406 in the cosolvent solution causes a drop in concentration after 48 hours of storage at room temperature (Table S3), but there is no loss of purity indicating R406 is chemically stable in the co-solvent solution for 48 hours at room temperature (Figure S4).

**Table S3.** Short-term stability of R406 in cosolvent formulation at room temperature

|  | Initial Concentration | Storage Time = 48 hr |
| --- | --- | --- |
| Average Concentration (µM) | 743.6 | 252.5 |
| Percentage of Initial Concentration | --- | 34% |


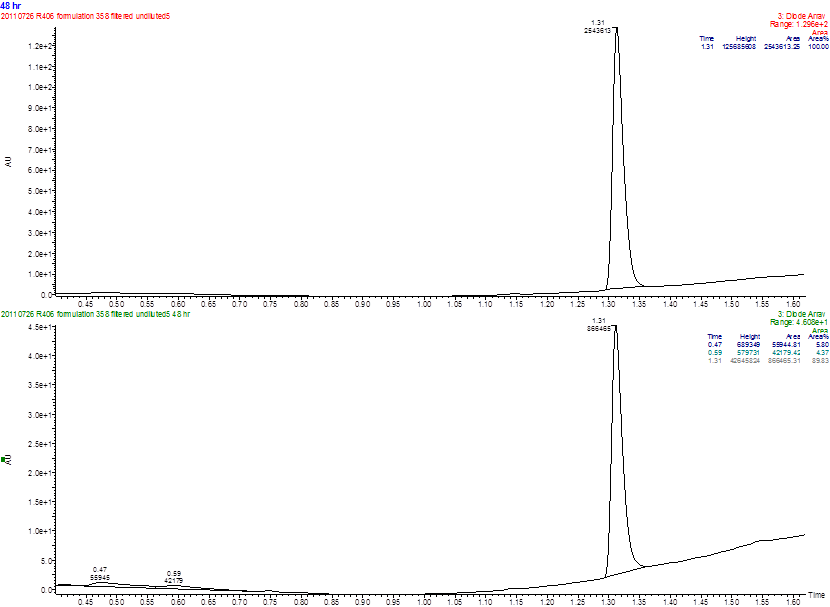


**Figure S2.** No loss of purity of R406 in cosolvent formulation after 48 hours storage at room temperature

**Mammalian cell drug susceptibility assay - methods**

RB355 retinoblastoma cells were maintained in RPMI medium (Lonza RPMI-1640) supplemented with 10% FBS (Equitech-Bio), penicillin, streptomycin, and glutamate (Gibco). BJ cells were grown in EMEM (Lonza) supplemented with 10% FBS, penicillin and streptomycin. 293T cells were grown in DMEM supplemented with 10% FBS, penicillin, streptomycin, and glutamate. Cells were passaged every 3-4 days or when they reached 70%-80% confluence.

RB335 cells were seeded in 6-well dishes. Seeded cells were incubated overnight at 37 °C in a humidified 5% CO_2_ incubator. Cells were treated with DMSO alone, 1, 2.5, or 5 µM R406 and returned to the incubator. At 0, 6, 12, 18, 24, and 72 h, the drugged media was removed from a subset of samples (n=3 samples per cell line) and replaced with drug-free media, washing out the R406 for the remainder of the 72 h incubation. After 72 hours, cells were suspended and aliquoted into a 96 well plate assaying viability (50 µL of cells per well, each sample assayed in duplicate and averaged). Plates were equilibrated at room temperature for 20 min before addition of 50 μL Cell Titer Glo (Promega) to each well. Plates were shaken on an orbital shaker for 2 min at 500 rpm. Luminescence was read after 15 min on an Envision plate reader (Perkin Elmer). Dissociated cells were also stained for caspase and EdU.

**Mammalian cell drug susceptibility assay - results**

Figure S3 shows viability of RB355 cells treated with 1, 2.5 or 5 µM R406 in culture for varied exposure durations (0, 6, 12, 18, 24, and 72 h) determined based on the Cell Titre Glo luminescence assay and normalized to DMSO treated cells (100% cell viability). Cells treated for 0h were incubated 72h without exposure to R406 and without any media washout during the incubation period. Cells treated for 72h were incubated with R406 for 72h without any media washout during the incubation period. Cell viability data shows good agreement with caspase and EdU staining results (Figure 3F-J). At a concentration of 2.5 µM R406, cell viability is reduced to 50% between 24 and 48 hours of exposure. At a concentration of 5 µM R406, cell viability is reduced to 50% between 12 and 24 hours of exposure. In cells treated with 1 µM R406 for up to 24h, there is no reduction in cell viability compared with DMSO treated cells. Even at longer exposure times (48h and 72h), the decrease in viability of RB355 cells exposed to 1 µM R406 never exceeds 20% (Figure S3). Cell viability is slightly higher at all concentrations for 48h compared to 72h, possibly because media in the 72h treatment group is never washed out. RB355 are semi-adherent, so washing out may remove a small subset of non-adherent cells, particularly in drug-treated groups. When normalized to DMSO treated cells, this may cause a slight overestimate of total viability.


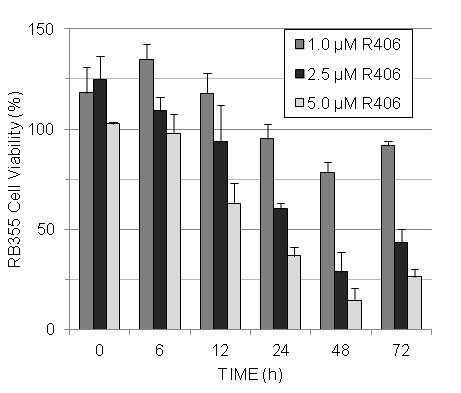


**Figure S3**. RB355 cells treated with 1, 2.5 or 5 µM R406 for varied exposure durations (0, 6, 12, 24, 48 or 72h). Cell viability is measured with Cell Titer Glo and normalized to DMSO-treated cells (100% cell viability).

**Stock Solutions**

Stock solutions of test compounds were prepared by dissolving R406 into DMSO to yield a concentration of 10 mM and by dissolving OSI-906 into acetonitrile to yield a concentration of 12 µM. Stock solutions were stored at room temperature. The working stock solutions (R406 at concentrations of 1 mM and 0.1 mM) were prepared by diluting the stock solution with DMSO.

**Quantitative Analysis of R406 in Mouse Plasma and Vitreous**

Chromatographic separation was performed on an Acquity UPLC BEH C18 1.7 µm spherical particle, 2.1 x 50 mm column (Waters Corporation, Milford, MA) using an Acquity ultra performance liquid chromatography (UPLC) system. The total flow rate was 1.0 mL/min. The sample injection volume was 10 µL. The UPLC column was maintained at 55 °C and the gradient program started at 90% A (0.1% formic acid in MilliQ H_2_O), changed to 70% A over 0.2 min, to 95 % B (0.1% formic acid in acetonitrile) over 1.4 minutes, held for 0.35 minutes, then to 90% A over 0.05 minutes. Mass spectrometry was performed on an Acquity UPLC/MS System (Waters Corporation, Milford, MA) equipped with a single-quadrapole mass spectrometer. The mass spectrometer was operated in positive-ion mode with electrospray ionization. The conditions were as follows: capillary voltage 3.4 kV, cone voltage 30 V, source temperature 130 °C, desolvation temperature 400 °C, desolvation gas 800 L/h, cone gas 100 L/h. Data were acquired using Masslynx v. 4.1 and analyzed using the Quanlynx software suite. A full scan range from m/z = 110-1000 in 0.2 s was used to acquire MS data. Single ion recording mass spectrometry ([M+H]^+^ ion m/z 471.4 for R406 and [M+H]^+^ ion m/z 422.5 for OSI-906) was used to quantify relative amounts of the samples. Two calibration curves were made to quantitate levels of R406 in murine plasma and vitreous by preparing a serial dilution series (3-fold steps from 10 µM to 13.7 nM) from working solutions (0.1 mM DMSO solution of R406) diluted in blank murine plasma or vitreous. Separate quality control samples were prepared using the same methodology at R406 concentrations of 41.2, 370.4, and 3333.3 nM (see supplement). No interfering peak was detected in either murine plasma (Figure S5) or murine vitreous (Figure S7). The average recovery of R406 from plasma was 109% (Figure S6), and recovery from vitreous was 91.8% (Figure S8). Lower limit of quantitation (LLOQ) for plasma and vitreous was 13.7 nM. Accuracies for within-day and between-day precision ranged from 78.4% to 100.8% for plasma (Table S4) and from 83.4% to 108.8% for vitreous (Table S8). Extracted samples were stable for at least 48 hours (Tables S5 and S9). Spiked murine plasma and vitreous samples stored at -20^o^C (Tables S6 and S10) and -80^o^C (Tables S7 and S11) were stable for at least 72 hours and 14 days, respectively.

For each pharmacokinetic sample, protein precipitation (PP) was performed prior to analysis. For murine plasma samples, a total of 50 µL of plasma sample was placed in a 96-well analytical plate (300 µL maximum volume in each well), and added to 100 µL of 12 µM OSI-906 (internal standard) in 100% acetonitrile. The plate was sealed, shaken at 600 rpm for 10 minutes, and then centrifuged at 10,000x*g* for 20 min at 4°C. 100 µL of the supernatant was transferred to a new analytical 96-well plate and 10 µL was injected onto the LC–MS/MS system. For the murine vitreous samples, a total of 5 µL of vitreous sample was placed in a 384-well analytical plate (80 µL maximum volume in each well), and quenched by 45 µL of 12 µM OSI-906 (internal standard) in 100% acetonitrile. The plate was sealed, shaken at 600 rpm for 10 minutes, and then centrifuged at 10,000x*g* for 20 min at 4°C. 40 µL of the supernatant was transferred to a new analytical 384-well plate and 10 µL was injected onto the LC–MS/MS system. Detailed methods and results of assay validation (including linearity, lower limit of quantitation, intra-day and inter-day precision and accuracy, recovery, and stability) follow.

**Assay Validation- Methods**

**Linearity and lower limit of quantitation:** Two calibration curves for murine plasma or vitreous samples were analyzed during the validation process. The linear regression of R406 peak areas was weighted by 1/x^2^. The correlation coefficient (R) and coefficient of determination (R^2^) were used to evaluate the linearity of each calibration curve. The lower limit of quantitation (LLOQ) was defined as the lowest concentration in each calibration curve, which had a precision and accuracy within 30% and a signal/noise (S/N) ratio greater than 5.

**Accuracy, precision, and recovery:** The methods developed for the measurement of R406 in murine plasma and vitreous were cross-validated over 3 days by analysis of plasma quality control samples, and the intra-day and inter-day precision and accuracy for the method was determined. Recovery was assessed by preparing all seven calibrators in triplicate alongside neat samples of the same concentrations in 100% DMSO. Both were extracted and analyzed to assess percent recovery.

**Stability:** The extracted solution stability of R406 from murine plasma or vitreous was assessed at low (41.2nM), medium (370.4 nM), and high (3333.3 nM) concentrations in triplicate at room temperature. Blank murine plasma was spiked with the indicated concentrations of R406 and extracted solutions were stored at room temperature for day 0, and day 2. The short-term stability of R406 in murine plasma or vitreous at -20 °C was assessed at three concentrations in triplicate. Samples were thawed at day 1 and 2. At day 2, protein precipitation was performed and samples treated with internal standard/acetonitrile as described previously: for murine plasma samples, 50 µL of sample was quenched by addition of 100 µL of 5 mg/L OSI-906 (internal standard) in 100% acetonitrile, followed by plate sealing, shaking and centrifuging, transfer of 100 µL of supernatant injection of 10 µL of supernatant onto the LC–MS/MS system; for murine vitreous samples, 5 µL of sample quenched by addition of 45 µL of 5 mg/L OSI-906 (internal standard) in 100% acetonitrile, followed by plate sealing, shaking and centrifuging, transfer of 40 µL of supernatant injection of 10 µL of supernatant onto the LC–MS/MS system. Long term stability of R406 in murine plasma or vitreous at −80 °C was assessed. Unextracted spiked murine plasma or vitreous at low, medium, and high levels was frozen at −80 °C for 0, 7, and 14 days. Samples were thawed immediately extracted with acetonitrile/OSI-906, and quantitated. All samples were analyzed in triplicates.

**Assay Validation – Results**

No interfering peak was detected in either murine plasma (Figure S6) or murine vitreous (Figure S8). The average recovery of R406 from plasma was 109% (Figure S7), and recovery from vitreous was 91.8% (Figure S9). Lower limit of quantitation (LLOQ) for plasma and vitreous was 13.7 nM. Accuracies for within-day and between-day precision ranged from 78.4% to 100.8% for plasma (Table S4) and from 83.4% to 108.8% for vitreous (Table S8). Extracted samples were stable for at least 48 hours (Tables S5 and S9). Spiked murine plasma and vitreous samples stored at -20^o^C (Tables S6 and S10) and -80^o^C (Tables S7 and S11) were stable for at least 72 hours and 14 days, respectively.


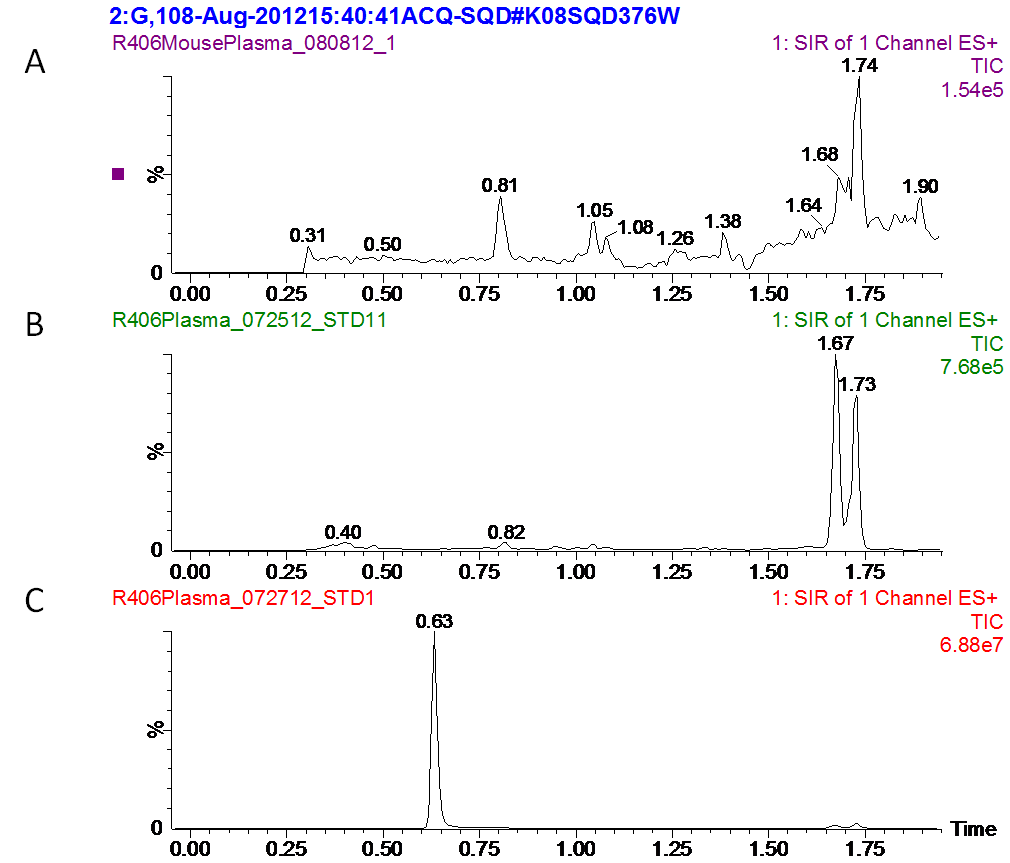


**Figure S6.** No interference peak in murine plasma. A. Blank individual mouse plasma extraction B. Blank pooled mouse plasma extraction C. Spiked plasma (HLOQ)


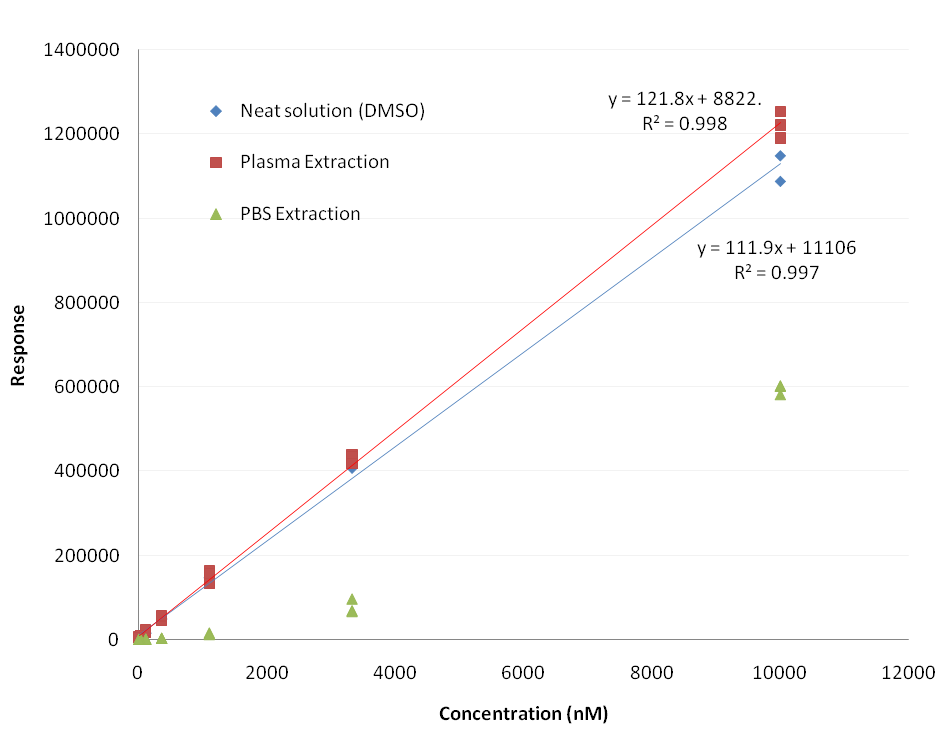


**Figure S7.**Recovery from murine plasma.

**Table S4.** Inter- and Intra-day Precision for R406 quantitation in murine plasma

|  |  | **Theoretical Conc (nM)** | **Average Read Conc. (nM)** | **Accuracy%** |
| --- | --- | --- | --- | --- |
| **Inter-day** | High Control | 3333.3 | 3088.8 ±74.1 | 92.7 |
|  | Middle Control | 370.4 | 330.3 ± 4.4 | 89.2 |
|  | Low Control | 41.2 | 41.5 ± 10.1 | 100.8 |
| **Intra-day** | High Control | 3333.3 | 2943.2 ± 140.4 | 88.3 |
|  | Middle Control | 370.4 | 290.5 ± 2.7 | 78.4 |
|  | Low Control | 41.2 | 35.7 ± 11.3 | 86.8 |

**Table S5.** Stability of extracted samples (R406 in murine plasma)

|  |  | **Theoretical Conc (nM)** | **Average Read Conc. (nM)** | **Accuracy%** |
| --- | --- | --- | --- | --- |
| **Inject at Day 1** | High Control | 3333.3 | 3088.8± 74.1 | 92.7 |
|  | Middle Control | 370.4 | 330.3± 4.4 | 89.2 |
|  | Low Control | 41.2 | 41.5± 10.1 | 100.8 |
| **Injected at Day 3 (Extracted samples in autosampler for 2 days)** | High Control | 3333.3 | 3147.7± 153.1 | 94.4 |
|  | Middle Control | 370.4 | 361.5± 46.3 | 97.6 |
|  | Low Control | 41.2 | 40.1± 9.5 | 97.4 |

**Table S6.** Short-term stability of R406 in murine plasma at -20^o^C

|  |  | **Theoretical Conc (nM)** | **Average Read Conc (nM)** | **Accuracy%** |
| --- | --- | --- | --- | --- |
| **Inject at Day 1** | High Control | 3333.3 | 3088.8 ± 74.1 | 92.7 |
|  | Middle Control | 370.4 | 330.3 ± 4.4 | 89.2 |
|  | Low Control | 41.2 | 41.5 ± 10.1 | 100.8 |
| **Injected at Day 3 (Samples thawed on Day 2)** | High Control | 3333.3 | 3185.7 ± 383.8 | 95.6 |
|  | Middle Control | 370.4 | 364.6 ± 31.9 | 98.4 |
|  | Low Control | 41.2 | 44.9 ± 5.3 | 109.2 |
| **Injected at Day 3 (Samples thawed on Day 3)** | High Control | 3333.3 | 3008.9 ± 303.5 | 90.3 |
|  | Middle Control | 370.4 | 375.9 ± 8.9 | 101.5 |
|  | Low Control | 41.2 | 39.5 ± 9.8 | 96.1 |

**Table S7.**Long-term stability of R406 in murine plasma at -80^o^C

|  |  | **Theoretical Conc (nM)** | **Average Read Conc. (nM)** | **Accuracy%** |
| --- | --- | --- | --- | --- |
| **Inject at Day 1** | High Control | 3333.3 | 3088.8 ± 74.1 | 92.7 |
|  | Middle Control | 370.4 | 330.3 ± 4.4 | 89.2 |
|  | Low Control | 41.2 | 41.5 ± 10.1 | 100.8 |
| **Injected at Day 8** | High Control | 3333.3 | 2810.6 ± 154.7 | 84.3 |
|  | Middle Control | 370.4 | 344.8 ± 21.5 | 93.1 |
|  | Low Control | 41.2 | 36.1 ± 3.2 | 87.8 |
| **Injected at Day 15** | High Control | 3333.3 | 2984.3 ± 96.8 | 89.5 |
|  | Middle Control | 370.4 | 313.7 ± 35.8 | 84.7 |
|  | Low Control | 41.2 | 34.9 ± 2.7 | 84.8 |

**
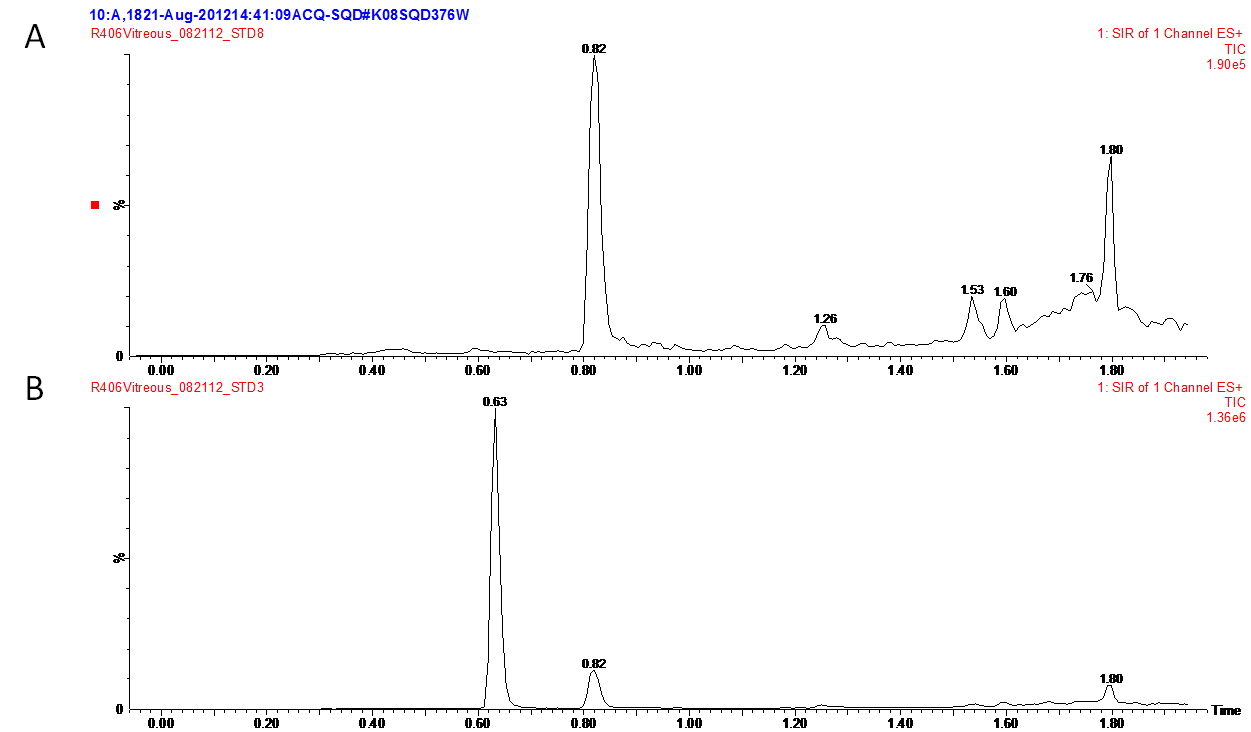
**

**Figure S5.** No interference peak in murine vitreous. A. Blank pooled mouse vitreous extraction B. Spiked vitreous.


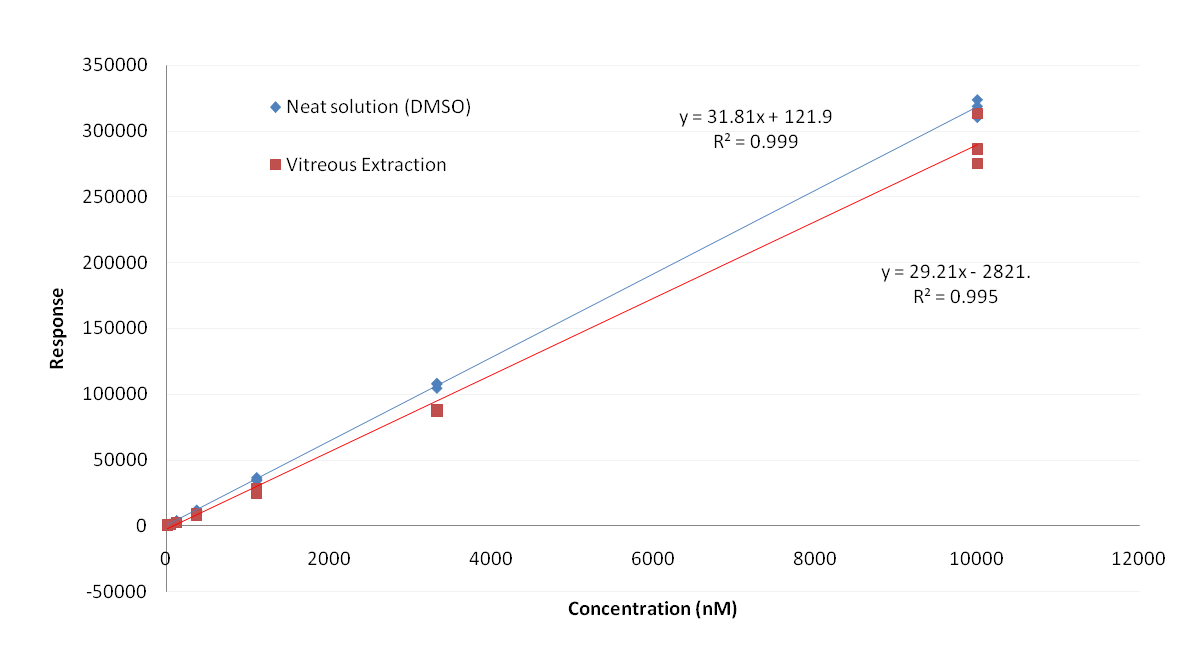


**Figure S7.** Recovery from murine vitreous.

**Table S8.** Inter- and Intra-day Precision for R406 quantitation in murine vitreous

|  |  | **Theoretical Conc (nM)** | **Average Read Conc. (nM)** | **Accuracy%** |
| --- | --- | --- | --- | --- |
| **Inter-day** | High Control | 3333.3 | 3149.4 ± 82.2 | 94.5 |
|  | Middle Control | 370.4 | 333.2 ± 54.2 | 90.0 |
|  | Low Control | 41.2 | 34.3 ± 1.8 | 83.4 |
| **Intra-day** | High Control | 3333.3 | 3070.0 ± 307.0 | 92.1 |
|  | Middle Control | 370.4 | 402.9 ± 103.0 | 108.8 |
|  | Low Control | 41.2 | 40.8 ± 9.1 | 99.2 |

**Table S9.** Stability of extracted samples (R406 in murine vitreous)

|  |  | **Theoretical Conc (nM)** | **Average Read Conc. (nM)** | **Accuracy%** |
| --- | --- | --- | --- | --- |
| **Inject at Day 1** | High Control | 3333.3 | 3149.4 ± 82.2 | 94.5 |
|  | Middle Control | 370.4 | 333.2 ± 54.2 | 90.0 |
|  | Low Control | 41.2 | 34.3 ± 1.8 | 83.4 |
| **Injected at Day 3 (Extracted samples in autosampler for 2 days)** | High Control | 3333.3 | 3053.7 ± 103.5 | 91.6 |
|  | Middle Control | 370.4 | 328.9 ± 8.0 | 88.8 |
|  | Low Control | 41.2 | 39.5 ± 7.9 | 96.0 |

**Table S10.**Short-term stability of R406 in murine vitreous at -20^o^C

|  |  | **Theoretical Conc (nM)** | **Average Read Conc. (nM)** | **Accuracy%** |
| --- | --- | --- | --- | --- |
| **Inject at Day 1** | High Control | 3333.3 | 3149.4 ± 82.2 | 94.5 |
|  | Middle Control | 370.4 | 333.2 ± 54.2 | 90.0 |
|  | Low Control | 41.2 | 34.3 ± 1.8 | 83.4 |
| **Injected at Day 3 (Samples thawed on Day 2)** | High Control | 3333.3 | 3038.1 ± 376.5 | 91.1 |
|  | Middle Control | 370.4 | 345.3 ± 10.2 | 93.2 |
|  | Low Control | 41.2 | 45.0 ± 2.1 | 109.4 |
| **Injected at Day 3 (Samples thawed on Day 3)** | High Control | 3333.3 | 3009.2 ± 343.4 | 90.3 |
|  | Middle Control | 370.4 | 349.6 ± 5.8 | 94.4 |
|  | Low Control | 41.2 | 39.8 ± 10.4 | 96.6 |

**Table S11.** Long-term stability of R406 in murine vitreous at -80^o^C

|  |  | **Theoretical Conc (nM)** | **Average Read Conc. (nM)** | **Accuracy%** |
| --- | --- | --- | --- | --- |
| **Inject at Day 1** | High Control | 3333.3 | 3411.1 ± 254.4 | 102.3 |
|  | Middle Control | 370.4 | 360.8 ± 60.7 | 97.4 |
|  | Low Control | 41.2 | 41.3 ± 1.9 | 100.4 |
| **Injected at Day 8** | High Control | 3333.3 | 3214.4 ± 210.5 | 96.4 |
|  | Middle Control | 370.4 | 324.1 ± 23.4 | 87.5 |
|  | Low Control | 41.2 | 32.4 ± 3.1 | 78.7 |
| **Injected at Day 15** | High Control | 3333.3 | 3508.2 ± 193.8 | 105.2 |
|  | Middle Control | 370.4 | 368.3 ± 25.7 | 99.4 |
|  | Low Control | 41.2 | 43.4 ± 7.1 | 105.5 |

Emulsion Stability

An emulsion of 30% castor oil, 5% Cremaphor, 4% T80, 61% PBS was prepared and samples were stored at 4^o^C and room temperature (25^o^C). Samples were diluted and OD_600_ for each dilution was measured in triplicate on the day of emulsion preparation (storage time = 0 days) and after 6 days of storage. OD_600_ was measured using a black Costar clear bottom plate and a Spectramax plate reader. No change in absorbance was observed after 6 days of storage (see Table S12 and Figure S10).

R406 free base stored in castor oil at room temperature was stable at least 6 days (average concentration of R406 remaining after 6 days was 113.3% of the initial concentration, n =3). R406 stored in castor oil emulsion at room temperature was stable at least 72 hours (see Table S13). For storage stability in emulsion, n =4.

**Table S12.** Emulsion Stability

| **Emulsion**  **Dilution** | **Initial absorbance (OD_600_)** | **Absorbance (OD_600_) after 6 days storage** |
| --- | --- | --- |
| 1 in 10 | 2.03 ± 0.005 | 2.04 ± 0.005 |
| 1 in 20 | 1.91 ± 0.003 | 1.91 ± 0.006 |
| 1 in 40 | 1.74 ± 0.007 | 1.75 ± 0.002 |
| 1 in 80 | 1.51 ± 0.021 | 1.51 ± 0.003 |
| 1 in 160 | 1.17 ± 0.013 | 1.19 ± 0.001 |
| 1 in 320 | 0.78 ± 0.007 | 0.80 ± 0.001 |
| 1 in 640 | 0.45 ± 0.007 | 0.48 ± 0.006 |
| 0 (buffer alone) | 0.039 ± 0.000 | 0.039 ± 0.000 |


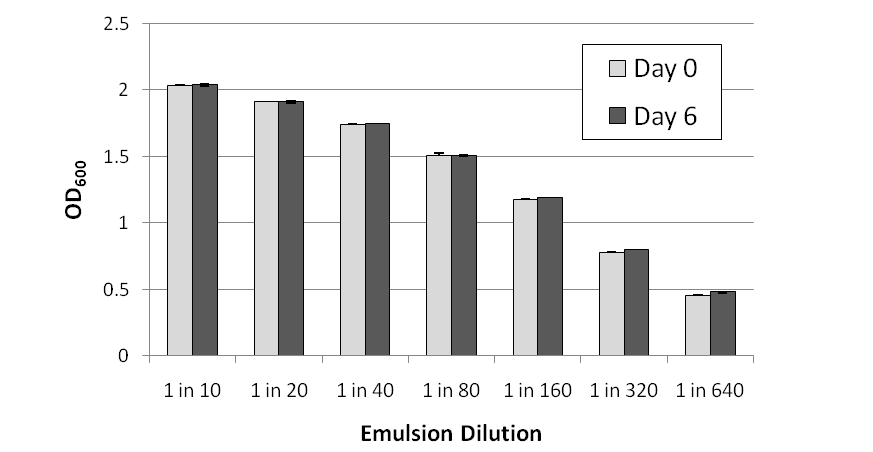


**Figure S10.** Emulsion stability (6 days storage at room temperature)

**Table S13.** R406 Stability in Castor Oil Emulsion at Room Temperature

|  | Initial Concentration | Storage Time = 24 hr | Storage Time = 48 hr | Storage Time = 72 hr |
| --- | --- | --- | --- | --- |
| Average Concentration (mM) | 12.39 ± 1.21 | 11.03 ± 2.09 | 9.78 ± 3.13 | 12.45 ± 1.43 |
| Percentage of Initial Concentration | --- | 89.0% | 78.9% | 100.5% |

**Table S14**. R406 phenylsulfonate salt and free base crashing upon dilution of DMSO stock in PBS

|  | Theoretical Concentration | Measured Concentration |
| --- | --- | --- |
| PBS + 5% HPβCD unfiltered | 0 | 0 |
| PBS + 5% HPβCD, 0.22 micron filtered | 0 | 0 |
| R406 phenylsulfonate salt 10 mM DMSO stock | 10 mM | 10.82 mM |
| R406 free base 10 mM DMSO stock | 10 mM | 11.14 mM |
| R406 phenylsulfonate salt stock diluted 1 in 10 in PBS prior to filtration | 1.08 µM | 1.01 mM |
| R406 phenylsulfonate salt DMSO stock diluted 1 in 20 in PBS prior to filtration | 541 µM | 559 µM |
| R406 free base DMSO stock diluted 1 in 10 in PBS prior to filtration | 1.11 mM | 965 µM |
| R406 free base DMSO stock diluted 1 in 10 in PBS prior to filtration | 557 µM | 510 µM |
| R406 phenylsulfonate salt DMSO stock diluted 1 in 10 in PBS, 0.22 micron filtered | 1.01 mM | 88 µM |
| R406 phenylsulfonate salt DMSO stock diluted 1 in 20 in PBS, 0.22 micron filtered | 559 µM | 98 µM |
| R406 free base DMSO stock diluted 1 in 10 in PBS, 0.22 micron filtered | 965 µM | 7 µM |
| R406 free base DMSO stock diluted 1 in 20 in PBS 0.22 micron filtered | 510 µM | 6 µM |
